# Functioning of a Fluorescein pH-Probe in Aqueous Media: Impact of Temperature and Viscosity

**DOI:** 10.3390/mi14071442

**Published:** 2023-07-18

**Authors:** Darya P. Surzhikova, Lev A. Sukovatyi, Elena V. Nemtseva, Elena N. Esimbekova, Evgenia A. Slyusareva

**Affiliations:** 1 Institute of Engineering Physics and Radioelectronics, Siberian Federal University, 660041 Krasnoyarsk, Russia; dsurzhikova@sfu-kras.ru; 2 Biophysics Department, Siberian Federal University, 660041 Krasnoyarsk, Russia; lsukovatyy@sfu-kras.ru (L.A.S.); enemtseva@sfu-kras.ru (E.V.N.); esimbekova@yandex.ru (E.N.E.); 3 Institute of Biophysics, Siberian Branch of Russian Academy of Sciences, 660036 Krasnoyarsk, Russia

**Keywords:** fluorescein, chemosensor, ratiometric signal, protolytic equilibrium, viscosity, steady-state fluorescence, molecular dynamics, preferential interaction, temperature effect, hydrogen bond

## Abstract

In this work, we considered the influence of viscogenic agents (glycerol, sucrose) as well as the temperature on the fluorescent characteristics of fluorescein at pH 6.5 in order to describe the acid-base status of local environment in terms of a spectrally detectable dianion-anion equilibrium. The protolytic equilibrium of fluorescein was found to depend on the solvent viscosity in a complex way. Whereas in the presence of sucrose the ratiometric signal of fluorescein (I^488^/I^435^) remains rather unchanged, the addition of glycerol (up to 40% *w*/*w*) results in the increase of the signal (up to 19%), that can be attributed to the different mechanisms of cosolvents effects on dye molecules in the ground state. Molecular dynamics of the dye in the presence of glycerol and sucrose revealed that the cosolvents preferentially interact with fluorescein monoanion and dianion, displacing water molecules from the local environment which in turn reduces the average number of the hydrogen bonds between xanthene ring of the dye and water molecules. The ratiometric signal demonstrates linear growth with the temperature in the range of 10–80 °C regardless of the presence of viscogenic agents. A linear correlation between the temperature sensitivity of the ratiometric signal and the change in the molar enthalpy of the proton dissociation reaction in buffer and viscous media was determined.

## 1. Introduction

Small-molecular fluorescent probes find various applications at comprehensive investigation of physical-chemical characteristics of inhomogeneous media providing both spatial and temporal resolution [1]. Thus, an intensive green luminescence of fluorescein as well as its derivative fluorescein isothiocyanate is widely used in biomedical research for the labeling of proteins and other macromolecules [2,3,4].

**Figure 1:**
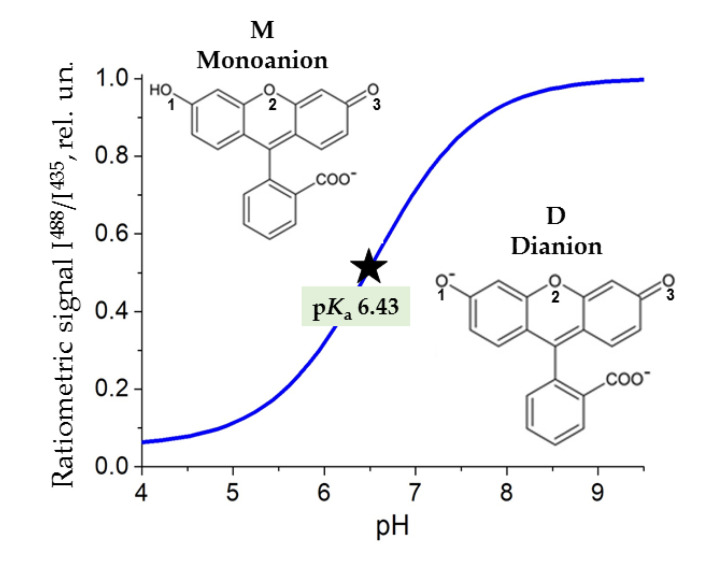
Ratiometric signal of the fluorescein vs. pH of the medium. I^488^ and I^435^ are integral fluorescence intensities under excitation at 488 and 435 nm, respectively. The position of p*K*_a_ [5] of monoanion-dianion equilibrium is indicated. The structures of monoanion and dianion of the fluorescein are shown. The numeration of oxygen atoms of the xanthene rings is indicated.

Along with bright luminescence, fluorescein has a protolytic equilibrium between dianionic (D) and monoanionic (M) forms in physiologically relevant pH range (p*K_a_* 6.43 [5]). D and M differ in their quantum yields by a factor of ~2.5 [5,6]. The ratio of I^488^/I^435^, shown in Figure 1, reflects the relation between the fluorescence intensities obtained at two different wavelengths providing preferential excitation of the dianionic (488 nm) or monoanionic (435 nm) forms (“ratiometric signal”). The value of I^488^/I^435^ demonstrates high sensitivity, reversibility and robustness to undesirable variations in probe concentration and instrumental performance. It is widely used in analytical techniques to determine pH [1,4].

When fluorescein is used as an intracellular probe, it has to function in viscous media that are densely packed with molecules of various sizes and chemical properties [7]. Under these conditions, the analytical signal from a microenvironment-sensitive probe cannot be interpreted solely in terms of pH, and the additional effects on protolytic equilibria must also be taken into account. It was shown that the equilibrium is substantially disturbed by the polarity of the media [8,9], the lipid-water interface of micelles and bilayers [10,11], or conjugation to macromolecules [12,13,14]. Previously, we investigated the impact of phosphate-ions on the ratiometric signal of fluorescein [15]. It was found that variation of the concentration of proton acceptors up to 1 M could shift equilibria of the chemosensor in both ground and excited states. This allows the fuorescein to fully exhibit its photoacidic properties. All mentioned factors may play a significant role both in nature (e.g., cells) and man-made systems [16,17]. 

The present study was focused on the investigation of the sensitivity of the ratiometric fluorescence response of fluorescein to the viscosity and temperature of the medium. These factors are known to modulate the rates of (bio)chemical reactions and overall cell metabolism [18]. Thus, the development of methods capable of determining intracellular viscosity and temperature is a demanding task [19]. We varied the viscosity of the solution from 1 to about 5 cP by addition of different concentrations of glycerol and sucrose, which brings the conditions closer to those observed for cells [20]. Additionally, we studied fluorescein properties under variation of the temperature in the range of 10–80 °C. In the presented experiments, pH of fluorescein solution was fixed at a value of 6.5, which is close to the inflection point of the ratiometric signal. Under these conditions, only two ionic forms with comparable proportions should be considered. We used a combination of steady-state fluorescence spectroscopy and molecular dynamics techniques to reveal the mechanisms and magnitude of the ratiometric signal perturbation to varying probe environment conditions. The results obtained can increase the information content of ratiometric fluorescence signals, allowing separate consideration of factors of different nature that affect fluorescence. This approach is useful for interpretation of ratiometric data during fluorescein probe application in both biological/biomedical and synthetic/industrial systems, including biochemical reactors and microfluidic devices.

## 2. Materials and Methods

### 2.1. Chemicals

Fluorescein (Sigma-Aldrich, St. Louis, MI, USA, quality level 200), glycerol (MP Biomedicals, LLC, Irvine, CA, USA, purity > 99%), sucrose (Panreac, Barcelona, Spain, USP-NF Grade) were used without further purification. Phosphate buffer components–distillate water solutions of K_2_HPO_4_ (Sigma-Aldrich, St. Louis, MI, USA, purity > 99%) and NaH_2_PO_4_ (Sigma-Aldrich, purity > 99%)–were initially prepared with concentration of 2 M. The ratios of the two components were varied to obtain pH 6.5 and pH 5.0. The viscosity of the medium was changed by addition of glycerol (0–50% *w*/*w*) or sucrose (0–37% *w*/*w*) to phosphate buffer so that the final concentration of phosphate ions were 1 M. Reference solution of fluorescein dianion was prepared at pH 11.0 by titration of water with KOH. The concentration of the fluorescein was 8 μM in all samples. All measurements were performed 60 min after preparing the solution.

The viscosity of the solutions was calculated based on the concentration of the viscogenic agents (glycerol, sucrose) [21,22]. The pH value of the solution was controlled using a Mettler Toledo SevenCompact S220 pH meter (Greifensee, Switzerland) with an error of 0.01.

### 2.2. Instruments and Experimental Procedure

Absorption spectra were obtained using spectrophotometer Carry 5000 (Agilent Technologies, Mulgrave, Australia).

Emission spectra were measured on a Fluorolog 3–22 spectrofluorometer (Horiba Jobin Yvon, Edison, NJ, USA) with excitation at 488 and 435 nm to excite predominantly dianion and monoanion of the fluorescein respectively. To make the value of the ratiometric signal independent of the experimental setup, we used a correction factor equal to the ratio of the xenon lamp intensities at the wavelengths. The spectra were corrected for reabsorption and the detector’s spectral sensitivity. All measurements were made using a 1 × 1 cm cuvette in the L-excitation geometry.

The temperature during spectral measurements was maintained using a thermostated cells of the instruments and directly controlled using a sensor (Hanna, Smithfield, RI, USA) with an accuracy of 0.1 °C.

### 2.3. Data Processing

**Figure 2:**
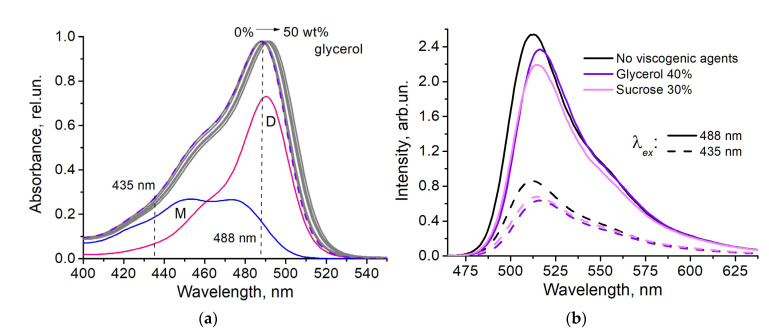
Spectral properties of the fluorescein in viscous media at pH 6.5: (**a**) absorption spectra in the presence of 0–50% glycerol (gray lines); an example of the decomposition of the spectrum without glycerol into monoanion (M, blue line) and dianion (D, pink line) components is shown, dashed violet line—fitted curve; (**b**) fluorescence spectra in buffer (black lines) and in the presence of 30% sucrose (pink lines) and 40% glycerol (violet lines) at excitation wavelengths 435 nm (dashed lines) and 488 nm (solid lines).

The equilibrium constant was calculated using the formula:

(1) $$K_{a}=\frac{\left[D\right]\times \left[H^{+}\right]}{\left[M\right]},$$

where [*D*] and [*M*] are the concentrations of dianion and monoanion respectively. The decomposition of the absorption spectra of fluorescein at pH 6.5 into *D* and *M* components (Figure 2a) was performed using Levenberg-Marquardt nonlinear approximation algorithm (OriginPro 8.0 software). To do this, we used the reference absorption spectra of *M* and *D*, which were obtained at pH 5.0 and 11.0 respectively under the relevant viscosity and temperature of the medium. Although the concentration of monoanions reaches a maximum at pH 5 the quota of fluorescein dianions and zwitterions remains about 3% and 4% respectively [6]. To consider the presence of the mentioned fluorescein forms we corrected the measured absorption spectra at pH 5.0 by subtracting the corresponding spectral contributions prior to the further data processing. The molar extinction coefficients [6] were used to recalculate the amplitudes of the spectra to [*D*] and [*M*]. The quality of fitting (R^2^) was ≥0.998.

The wavenumbers of 0-0 transitions of *M* and *D* were calculated as a half-sum of wavenumbers for the maxima of absorption and fluorescence spectra at pH 5.0 (for *M*) and 11.0 (for *D*).

### 2.4. Molecular Dynamics Simulations

Classical molecular dynamics (MD) of monoanion and dianion of the fluorescein surrounded by water molecules or a mixture of water with glycerol (40% *w*/*w*)/sucrose (30 % *w*/*w*) was performed using GROMACS 2020.4 [23]. The three-dimension structure of dianion was taken from PubChem database [24]. The structure of the monoanion was obtained by adding a hydrogen atom to O_1_ of the dianion using Chimera 1.15 [25]. OPLSA-AA force filed was used to describe atom-atom interaction in all modeled systems [26]. The topology files of both fluorescein forms were obtained from LigParGen web-based service [27]. Molecules of glycerol and sucrose were parameterized according to [28] and [29] respectively.

MD simulations were conducted for 100 ns in each explicit solvent with three independent runs. H-bonds between oxygens of the xanthene ring and solvent molecules were estimated by GROMACS gmx hbond plugin. The number of hydrogen bonds at each MD frame was defined as the number of donor-acceptor pairs satisfying the following geometric criteria: the acceptor-donor-hydrogen triplet angle ≤ 30° and the acceptor-donor distance ≤ 0.35 nm [23].

The spatial organization of water and cosolvents molecules in hydration layers of fluorescein were estimated by a minimum-distance distribution functions (MDDF) using the ComplexMixtures module in the Julia 1.8.2 software. The preferential interaction coefficients Γ for water, glycerol, and sucrose molecules were calculated based on MDDF [30].

## 3. Results

### 3.1. Spectral Properties of Fluorescein at pH 6.5 under Variation of Viscosity and Temperature of the Medium

We measured the absorption and fluorescence spectra of fluorescein in solutions with different viscosities (up to 4.2 cP with sucrose and to 5.4 cP with glycerol) and at varying temperatures (10–80 °C). Due to high cross-sensitivity of fluorescein dyes equilibrium to ionic strength [15,31], special effort was taken to fix the pH (6.5) and concentration of phosphate ions (1 M) of solutions with added viscogenic agents.

It was found that in viscous media the absorption spectra retained their shape and shifted bathochromically: by 4 nm in media with the addition of glycerol (50%), and by 1 nm in media with the addition of sucrose (37%) (Figure 2a). Under the conditions used (pH 6.5), the fluorescein solution contains two components, the dianionic and monoanionic forms with specific absorption bands (Figure 2a).

The effect of viscogenic agents on fluorescence spectra obtained at excitation wavelengths of 435 and 488 nm was manifested in a bathochromic shift and a decrease in intensity (Figure 2b). The profiles of the fluorescence spectra at the two excitation wavelengths are not identical sice different ionic forms are predominantly excited.

**Figure 3:**
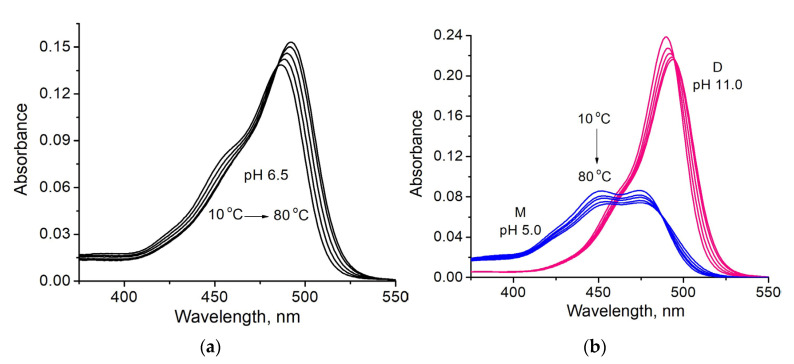
Absorption spectra of the fluorescein in buffers at different temperatures (10–80 °C): (**a**) at pH 6.5 (M + D); (**b**) at pH 5 (M, blue lines) and 11 (D, pink lines).

The increase in the temperature of the fluorescein solution at pH 6.5 led to the bathochromic shift of absorption spectra and the apparent increase of peak absorption (Figure 3a). However, study of the absorption spectra of the individual fluorescein forms (M at pH 5 and D at pH 11) revealed that decreasing extinction under heating was observed (Figure 3b). This suggests that observed spectral changes of the fluorescein at pH 6.5 are not the sum of the temperature effects on two ionic components and some chemical mechanisms are involved as well.

**Figure 4:**
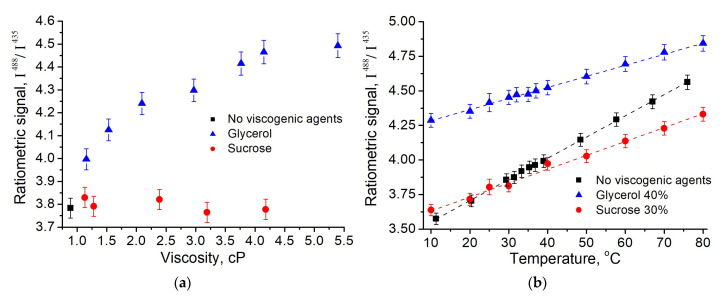
Dependencies of the ratiometric signal I^488^/I^435^ of the fluorescein on the viscosity (25 °C) (**a**) and temperature (**b**) of the media (pH 6.5) in the presence of glycerol (blue symbols) and sucrose (red symbols). Black symbols refer to the data obtained without viscogenic agents.

The change of the absorption and fluorescence properties of the fluorescein In solutions with various viscosity and temperature could change the ratiometric signal of this probe, which is used as indicator of the medium pH. The analysis confirms that it is true in most of the cases under consideration (Figure 4).

The viscosity dependencies revealed that in the presence of glycerol ratiometric signal increased non-linearly (with a maximum change of 19%), while in the presence of sucrose it remained the same as in the absence of the cosolvents (Figure 4a). The heating of the solutions causes the linear increase of the ratiometric signal, both in presence and in absence of the viscogenic agents (Figure 4b). The temperature dependencies were found to be reproduced in cooling, and their sensitivity to temperature is different for the studied media (Table 1).

### 3.2. Ground State Equilibrium of the Fluorescein at pH 6.5 under Variation of Viscosity and Temperature of the Medium

**Figure 5:**
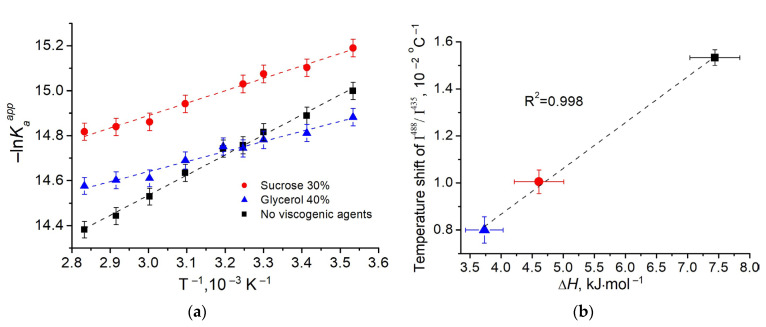
(**a**) The van’t Hoff plot for protolytic equilibrium of the fluorescein in buffer (black symbols) and in the presence of sucrose (30%) (red symbols) and glycerol (40%) (blue symbols) at pH 6.5. (**b**) Temperature sensitivity of the ratiometric signal vs. change of the enthalpy of reaction in the presence and absence of viscogenic agents. The coefficient of linear correlation R^2^ is shown. The dashed lines are linear approximations of the data.

Using decomposition of the absorption spectra of the fluorescein at pH 6.5 into the spectra of the monoanion and dianion we calculated the apparent ionic equilibrium constant $K_{a}^{app}$ for fluorescein in viscous media under different temperatures. A more rigorous evaluation of equilibrium constant requires taking into account the activity of the ions in the buffer solution [32]. We found that at 25 °C without cosolvents $pK_{a}^{app}$ = 6.45 ± 0.10, which is in good agreement with the published data [15]. $K_{a}^{app}$ was found to decrease under increasing temperature from 10 to 80 °C, with different slopes for buffer and viscous media with 30% sucrose and 40% glycerol (Table 1). In accordance with van’t Hoff equation, we analyzed the dependence −ln$K_{a}^{app}$(1/T) (Figure 5a) and determined the change in molar enthalpy of the reaction Δ*H* (Table 1). For buffer Δ*H* = 7.4 kJ·mol^–1^ was obtained, which is slightly lower than previously published value (9.6 kJ·mol^–1^ [32]) probably due to different buffer composition. The presence of viscogenic agents was found to reduce Δ*H* and the amplitude of the shift of $pK_{a}^{app}$ with the temperature increase (Table 1). Interestingly, at 25 °C the fluorescein in glycerol solution (40%) and in sucrose solution (30%) demonstrated $pK_{a}^{app}$ close to that in buffer. A similar result was obtained for fluorescein in a number of aqueous polymer solutions [33].

**Table 1:** Parameters of the temperature dependence of the ratiometric signal of the fluorescein I^488^/I^435^ and thermodynamic parameters of its equilibrium at pH 6.5.

Solvent (Buffer pH 6.5)	Temperature Shift of I^488^/I^435 #^, 10^−2^ °C^−1^	$p{K_{a}^{app}}^{\;\&}\;\mathrm{at}\;25\;{}^{\circ}C$	$\mathrm{Temperature}\;\mathrm{Shift}\;\mathrm{of}\;pK_{a}^{app},\;{}^{\circ}C{}^{-1}$	ΔH ^&^, kJ·mol^−1^	$\Delta p{K_{a}^{app}}^{\;\$}$
No viscogenic agents	1.53 ± 0.03	6.45 ± 0.10	–0.0039 ± 0.0001	7.4 ± 0.4	–0.71 ± 0.09
Sucrose (30%)	1.01 ± 0.05	6.55 ± 0.11	–0.0023 ± 0.0001	4.6 ± 0.4	–0.65 ± 0.08
Glycerol (40%)	0.80 ± 0.04	6.43 ± 0.09	–0.0019 ± 0.0001	3.7 ± 0.3	–0.69 ± 0.09

^#^ determined as a slope of dependencies in Figure 4b; ^&^ determined using van’t Hoff equation; ^$^ determined using Equation (2).

The observed distinction in the parameters of protolytic equilibrium for buffer and viscous media is attributed with a change of the microenvironment and hydration shell of the dianion and monoanion of the fluorescein. The correlation between the temperature sensitivity of the ratiometric signal and the change in the enthalpy of reaction in the presence of viscogenic agents was revealed (Figure 5b).

### 3.3. The Effects of Polarity of the Viscous Media

Ratiometric signal of the fluorescein depends on the absorption of the ionic forms at wavelengths 435 and 488 nm, at which the excitation of the fluorescence is carried out. Thus, the shift of the absorption spectra due to a change in the polarity of the medium or other reasons could lead to the disturbance of the I^488^/I^435^ even without any change of monoanion-dianion equilibrium. Addition of glycerol and sucrose to the buffer (water) solution do decrease the polarity of the medium. In this part of study, we aimed to estimate the influence of such decreasing trend observed in spectral properties of fluorescein.

The wavenumbers of the maximum of absorption spectra ν_abs_^m^ of the fluorescein in viscous media at pH 11, 6.5 and 5 were analyzed. The solvent polarity function or reaction field factor of the viscous media Δf(n, ε) was calculated according to the Lippert-Mataga equation [34].

**Figure 6:**
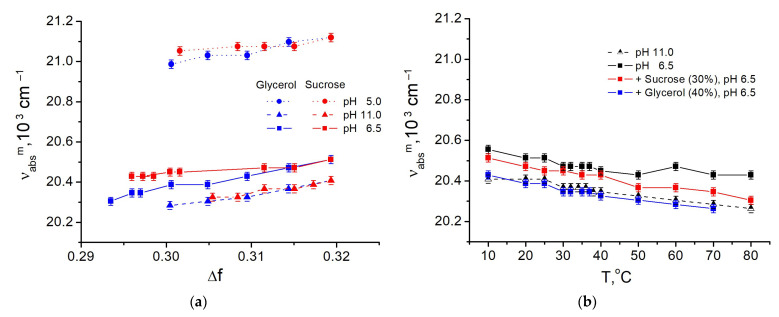
The wavenumber of absorption maximum ν*_abs_^m^* of fluorescein in viscous media with glycerol (blue) and sucrose (red) and without additives (black) at different pH: the dependencies on polarizability of the medium Δf (**a**) and on the temperature of the medium T (**b**).

We found that in the presence of the cosolvents (in media with lower polarity) the ν_abs_^m^ decreased for all of the samples: fluorescein monoanion (at pH 5), fluorescein dianion (at pH 11) and their mixture (at pH 6.5) (Figure 6a). At pH 5 and 6.5, the shift was less pronounced in the media with sucrose, which can be due to specific interaction of the cosolvent with monoanion. As a result, the difference in the ratiometric signals observed in the presence of sucrose and glycerol (Figure 4a) could be due to the different excitation efficiency at 435 nm of the monoanion of the fluorescein.

The temperature dependence of ν*_abs_^m^* also demonstrates bathochromic shift under heating (Figure 6b) for both dianion (at pH 11) and the monoanion-dianion mixture (at pH 6.5). For temperature effect, the change of the p*K_a_^app^* was revealed (Table 1), which means that the ratiometric signal does reflect the change of the monoanion-dianion equilibrium of the fluorescein, although this change is not related to the pH of the medium.

It is well understood that the decrease of polarity of the solvent causes a hypsochromic shift of absorption spectra of fluorescein, while the decrease of hydrogen bonding with the medium has the opposite effect [35]. Thus, in the spectral shifts we observed the results of action of two mechanisms and the change of H-bonding properties seems to dominate in the total effect. To clarify the specific interactions of fluorescein anions with the molecules of water, sucrose, and glycerol, we used molecular modeling techniques.

### 3.4. Interactions of Fluorescein with Sucrose and Glycerol Revealed by Molecular Dynamics

**Figure 7:**
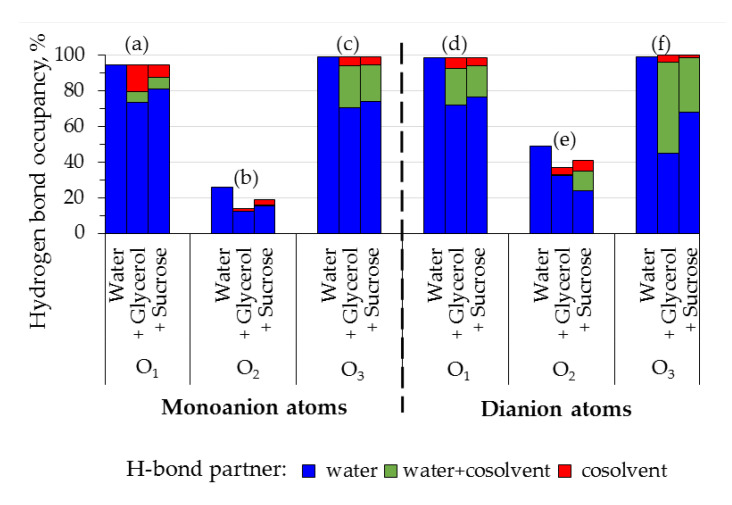
Occupancy of the hydrogen bonds between oxygen atoms O_1_, O_2_, and O_3_ (Figure 1) of xanthene ring of fluorescein monoanion (a–c) and dianion (d–f) and molecule(s) of water (blue), cosolvent (red) or both water and cosolvent (green). Occupancy was calculated as the fraction of simulation time during which H-bonds were determined in accordance with the geometrical criteria. Data for the models of fluorescein in water, glycerol 40% and sucrose 30% are shown.

The molecular dynamics of monoanion and dianion of fluorescein surrounded by water or mixtures of water with glycerol/sucrose was performed. The number of hydrogen bonds between the oxygens of the xanthene ring of fluorescein (O_1_, O_2_ and O_3_ in Figure 1) and the molecules of water and/or cosolvents was computed at each simulation step using geometrical criteria, as described in Section 2.4. The occupancy of hydrogen bond(s) was calculated as the fraction of simulation time during which H-bonds were determined (Figure 7).

The following patterns were revealed (Figure 7):

(i) Oxygens of the hydroxyl and carbonyl groups (O_1_ and O_3_) form bonds with water or/and cosolvents during 95–100% of the simulation time; the cosolvents partially replace the water molecules in hydrogen bonding with O_1_ and O_3_, but without a change in the total occupancy (sets (a), (c), (d), (f));
(ii) Heteroatom O_2_ is less available for hydrogen bonding (sets (b), (e)), and in the presence of cosolvents the total occupancy of its H-bonds decreases;
(iii) The cosolvents are more likely to form H-bonds with the dianion than the monoanion (compare contributions of the cosolvents in (a) and (d), (b) and (e), (c) and (f)).

Despite to the fact that O_1_ and O_3_ of the fluorescein dianion are supposed to have similar properties, they showed different capability to form hydrogen bonds with cosolvent molecules (Figure 7, sets (d) and (f)). O_3_ appeared to be more accessible for interactions with glycerol and sucrose than O_1_. 

**Figure 8:**
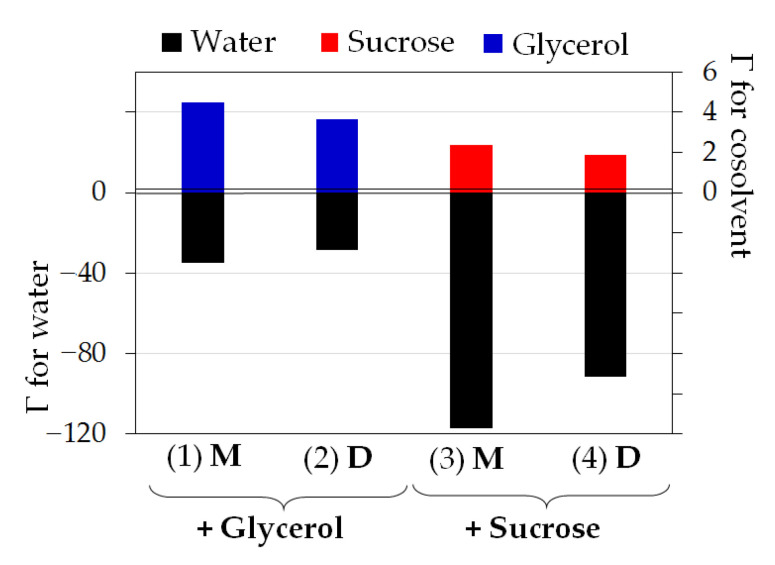
Coefficients of the preferential interactions Γ of water (black), glycerol (blue) and sucrose (red) with monoanion M (1 and 3) and dianion D (2 and 4) of the fluorescein.

Thus, the occupancy of the hydrogen bonds formed by xanthene ring of fluorescein with water decreases in the presence of glycerol and sucrose. This could be related to preferential interaction of the cosolvents with fluorescein, which was shown to contribute to solvatochromic effects on this dye [36]. To estimate the preferential interaction coefficients Γ for pairs M-water, M-cosolvent, D-water and D-cosolvent, we calculated the minimum-distance distribution function (MDDF) of cosolvent and water molecules relative to fluorescein mono- and di-anion [30]. Γ > 0 implies that concentration of the molecules near the solute is higher than in bulk solution, whereas Γ < 0 refers to the case when the molecules are excluded from the hydration shell of the solute [37]. Obtained values of Γ clearly indicated that water molecules were excluded from the surface of both monoanion and dianion in the presence of glycerol and sucrose, while the cosolvents showed preferential interaction with fluorescein (Figure 8). Sucrose is more effective in inducing water exclusion, but glycerol demonstrates slightly higher preferential interaction with fluorescein anions under conditions used.

The observed preferential interactions in triple system fluorescein-water-cosolvent indicates that the decrease of the solvent polarizability Δf used to analyze spectral shifts (Figure 7) could be underestimated: real concentration of the cosolvent near the fluorophore is higher than in bulk solution. Nevertheless, no hypsochromic shift of absorption was observed, but bathochromic was observed. This supports the conclusion about negligible contribution of the dispersion interactions into modulation of the spectral shifts of the fluorescein in protic solvents.

### 3.5. Excited State Equilibrium of the Fluorescein at pH 6.5 under Variation of Viscosity

Fluorescein is known to exhibit the properties of a photoacid, i.e., its pK_a_^*^ for monoanion-dianion equilibrium in the electronically excited state could substantially differ from that in the ground state [38,39]. To estimate this difference ($\Delta pK_{a}^{app}$) we used the Förster-Weller cycle:

(2) $$\Delta pK_{a}^{app}=hc\frac{{\tilde{\nu }}_{0-0}^{D}-{\tilde{\nu }}_{0-0}^{M}}{2.3kT},$$

where *k* is the Boltzmann constant, *c—*the speed of light in vacuum, *h—*Planck constant, ${\tilde{\nu }}_{0-0}^{D}$ and ${\tilde{\nu }}_{0-0}^{M}$ are the wavenumbers of 0-0–transitions for dianion and monoanion respectively.

We found that under conditions used (pH 6.5), $\Delta pK_{a}^{app}$ for fluorescein in sucrose solution (30%) and in glycerol solution (40%) reveal to be similar to that in buffer (−0.71 ± 0.09), (Table 1). Thus, the contribution of changes in the protolytic equilibrium of electronically excited fluorescein to the total effect of viscous media is insignificant.

## 4. Discussion

The application of the fluorescein in various pH-sensing techniques is usually based on the changes of its spectra associated with the monoanion-dianion equilibrium both in ground and in electronically excited states. The use of the two excitation wavelengths of 435 and 488 nm providing sufficient preferential absorption efficiency of mono- and dianionic forms of fluorescein respectively allows to register the ratiometric fluorescence signal I^488^/I^435^. A significant impact of both cosolvents and the temperature on the value of the measured ratiometric signal was revealed. Here, we discuss different mechanisms responsible for the observed variation of I^488^/I^435^ of the fluorescein at pH 6.5.

We found that cosolvents (glycerol and sucrose) preferentially interact with mono- and dianions of the fluorescein that leads to the partial displacement of water molecules from the local environment. Thus, the average number of the hydrogen bond between the dye ions and water reduces (Figure 7 and Figure 8). However, the enthalpy of the deprotonation decreases compared to the solution without organic cosolvents, which leads to the conclusion that the contribution of hydrophobic interactions with the cosolvent dominates over the hydrogen bonds contribution during the deprotonation of fluorescein monoanions. In the presence of sucrose (30%) this effect is more pronounced than in the presence of glycerol (40%) (Figure 8, Table 1). The role of the hydration shell of the dye for protolytic equilibrium of the fluorescein in the ground state was discussed in previous studies [10]. However, the hydrophobic contribution was rather underestimated.

Both the presence of the cosolvents (glycerol and sucrose) and heating were found to shift the absorption spectra of the dianion and monoanion of the fluorescein bathochromically (Figure 2 and Figure 3). This reduces the absorption efficiency of the ionic forms of the dye at the selected wavelengths compared to standard conditions (buffer solution, 25 °C). The effect of cosolvent is more pronounced at the excitation wavelength of 435 nm corresponding the range of the preferential monoanion absorption. Spectral shift can satisfactorily explain qualitative difference in ratiometric signal for sucrose and glycerol solutions under the same polarity of the solution medium (Figure 4a). However, the scale of the observed differences in the viscosity dependencies of I^488^/I^435^ suggests the consideration of some additional mechanisms. 

An additional factor, which can influence the ratiometric signal, is a change of the fluorescence quantum yield of the ionic forms due to lower polarity. The small deviation (by 2%) of quantum yield of the dianion in solutions of less polarity than water (methanol, ethanol, butanol and others) was reported [40]. Our data on the fluorescence intensity of fluorescein in viscous media at pH 11 correspond to those findings. For M the increase of quantum yield by 8–36% was reported in binary mixtures of water with acetonitrile, methanol, acetone and other less polar solvents [36]. The higher quantum yield of M would make the sensoric ratiometric signal less than it follows from the protolytic equilibrium.

Hypothetically we could expect a viscosity-controlled rate of proton transfer in the excited state of the fluorescein in viscous media due to the impact of the reorientational mobility of water [41], presence of hydrophobic groups of cosolvents [42] and water activity [43] on the proton mobility in aqueous solutions. The obtained data evidence that the changes in the ratiometric signal could be mainly explained by the processes in the ground state of the fluorescein. Thus, we revealed that the shift of the monoanion-dianion equilibrium of fluorescein in the excited state ($\Delta pK_{a}^{app}$) was not affected by the presence of the cosolvents. pK_a_^*^ remains biased toward dianion formation by about 0.7 as compared to the p*K_a_* in the ground state indicating that the mechanisms involving the charge redistribution in the excited state do not contribute to the dependence of the ratiometric signal on medium viscosity.

It should be emphasized that the ratiometric signal demonstrates a high linearity of the temperature dependency in the range of 10–80 °C regardless to the presence of viscogenic agents. A linear correlation of the temperature sensitivity of the ratiometric signal with the change in the molar enthalpy of the dissociation reaction reflects the independence of the latter parameter on the temperature (Figure 5b).

## 5. Conclusions

The mechanisms responsible for the sensitivity of the fluorescein ratiometric signal to the viscosity and temperature of the aqueous media under physiologically relevant pH (6.5) were investigated. Our findings indicate that specific effects of the sucrose and glycerol are mainly caused by their hydrophobic interactions with fluorescein ions reflecting in different shifts of the absorption spectra of the ionic forms of the dye. These shifts can be explained by changes in the polarity and/or hydrogen bonding status revealing a significant impact on the ratiometric signal. Thus, the effects of viscous media on the probe response were controlled rather by the nature of the viscogenic agents than by the value of the viscosity per se. Additionally, it was found that the presence of the viscogenic additives influences the thermal sensitivity of the ratiometric signal. Thus, the temperature-related shift of $pK_{a}^{app}$ of the monoanion-dianion equilibrium is less pronounced in a more viscous media indicating the decrease of the enthalpy of the proton transfer reaction. A high linearity of the temperature dependence of the ratiometric signal was observed. The findings can be further employed for the signal correction in fluorescein-based ratiometric analytical techniques.

## Data Availability

The data presented in this study are available on request from the corresponding author.

## References

[1] Kim H.N., Swamy K.M.K., Yoon J. (2011). Study on various fluorescein derivatives as pH sensors. Tetrahedron Lett..

[2] Hou X., Li Z., Li B., Liu C., Xu Z. (2018). An “off-on” fluorescein-based colormetric and fluorescent probe for the detection of glutathione and cysteine over homocysteine and its application for cell imaging. Sens. Actuators B Chem..

[3] Hungerford G., Benesch J., Mano J.F., Reis R.L. (2007). Effect of the labelling ratio on the photophysics of fluorescein isothiocyanate (FITC) conjugated to bovine serum albumin. Photochem. Photobiol. Sci..

[4] Lanz E., Gregor M., Slavík J., Kotyk A. (1997). Use of FITC as a Fluorescent Probe for Intracellular pH Measurement. J. Fluoresc..

[5] Sjöback R., Nygren J., Kubista M. (1995). Absorption and fluorescence properties of fluorescein. Spectrochim. Acta Part A Mol. Biomol. Spectrosc..

[6] Klonis N., Sawyer W.H. (1996). Spectral properties of the prototropic forms of fluorescein in aqueous solution. J. Fluoresc..

[7] Luby-Phelps K. (1999). Cytoarchitecture and physical properties of cytoplasm: Volume, viscosity, diffusion, intracellular surface area. Int. Rev. Cytol..

[8] Mchedlov-Petrossyan N.O., Tychina O.N., Berezhnaya T.A., Alekseeva V.I., Savvina L.P. (1999). Ionization and tautomerism of oxyxanthene dyes in aqueous butanol. Dyes Pigm..

[9] Mchedlov-Petrossyan N.O., Mayorga R.S. (1992). Extraordinary character of the solvent influence on protolytic equilibria: Inversion of the fluorescein ionization constants in H_2_O–DMSO mixtures. J. Chem. Soc. Faraday Trans..

[10] Kibblewhite J., Drummond C.J., Grieser F., Thistlethwaite P.J. (1989). Lipoidal eosin and fluorescein derivatives as probes of the electrostatic characteristics of self-assembled surfactant/water interfaces. J. Phys. Chem..

[11] Mchedlov-Petrossyan N.O., Kleshchevnikova V.N. (1994). Influence of the cetyltrimethylammonium chloride micellar pseudophase on the protolytic equilibria of oxyxanthene dyes at high bulk phase ionic strength. J. Chem. Soc. Faraday Trans..

[12] Sjöback R., Nygren J., Kubista M. (1998). Characterization of fluorescein-oligonucleotide conjugates and measurement of local electrostatic potential. Biopolymers.

[13] Talavera E.M., Alvarez-Pez J.M., Ballesteros L., Bermejo R. (1997). Fluorescein-Labeled DNA Probes for Homogeneous Hybridization Assays: Application to DNA *E. coli* Renaturation. J. Appl. Spectrosc..

[14] Friedrich K., Woolley P. (1988). Electrostatic potential of macromolecules measured by pKa shift of a fluorophore. 1. The 3’ terminus of 16S RNA. Eur. J. Biochem..

[15] Surzhikova D.S., Gerasimova M.A., Slyusareva E.A. (2022). Effect of Phosphate Ions on the Dianion–Anion Equilibrium of Fluorescein Excited State. Bull. Russ. Acad. Sci. Phys..

[16] Petty H.R. (2007). Fluorescence microscopy: Established and emerging methods, experimental strategies, and applications in immunology. Microsc. Res. Tech..

[17] Jain P., Aida T., Motosuke M. (2021). Fluorescence Anisotropy as a Temperature-Sensing Molecular Probe Using Fluorescein. Micromachines.

[18] Persson L.B., Ambati V.S., Brandman O. (2020). Cellular control of viscosity counters changes in temperature and energy availability. Cell.

[19] Yin J., Huang L., Wu L., Li J., James T.D., Lin W. (2021). Small molecule based fluorescent chemosensors for imaging the microenvironment within specific cellular regions. Chem. Soc. Rev..

[20] Puchkov E.O. (2013). Intracellular viscosity: Methods of measurement and role in metabolism. Biochem. Mosc. Suppl. A Membr. Cell Biol..

[21] Cheng N.S. (2008). Formula for the Viscosity of a Glycerol−Water Mixture. Ind. Eng. Chem. Res..

[22] Telis V.R.N., Telis-Romero J., Mazzotti H.B., Gabas A.L. (2007). Viscosity of Aqueous Carbohydrate Solutions at Different Temperatures and Concentrations. Int. J. Food Prop..

[23] Van Der Spoel D., Lindahl E., Hess B., Groenhof G., Mark A.E., Berendsen H.J. (2005). GROMACS: Fast, flexible, and free. J. Comp. Chem..

[24] Kim S., Chen J., Cheng T., Gindulyte A., He J., He S., Li Q., A Shoemaker B., A Thiessen P., Yu B. (2023). PubChem 2023 update. Nucleic Acids Res..

[25] Pettersen E.F., Goddard T.D., Huang C.C., Couch G.S., Greenblatt D.M., Meng E.C., Ferrin T.E. (2004). UCSF Chimera—A visualization system for exploratory research and analysis. J. Comp. Chem..

[26] Robertson M.J., Tirado-Rives J., Jorgensen W.L. (2015). Improved Peptide and Protein Torsional Energetics with the OPLS-AA Force Field. J. Chem. Theory Comput..

[27] Dodda L.S., Cabeza de Vaca I., Tirado-Rives J., Jorgensen W.L. (2017). LigParGen web server: An automatic OPLS-AA parameter generator for organic ligands. Nucleic Acids Res..

[28] Jahn D.A., Akinkunmi F.O., Giovambattista N. (2014). Effects of temperature on the properties of glycerol: A computer simulation study of five different force fields. J. Phys. Chem..

[29] Jamali S.H., Westen T.V., Moultos O.A., Vlugt T.J. (2018). Optimizing nonbonded interactions of the OPLS force field for aqueous solutions of carbohydrates: How to capture both thermodynamics and dynamics. J. Chem. Theory Comp..

[30] Martínez L. (2022). ComplexMixtures. jl: Investigating the structure of solutions of complex-shaped molecules from a solvent-shell perspective. J. Mol. Liq..

[31] Schröder C.R., Weidgansa B.M., Klimant I. (2005). pH Fluorosensors for use in marine systems. Analyst.

[32] Smith S.A., Pretorius W.A. (2002). Spectrophotometric determination of pK_a_ values for fluorescein using activity coefficient corrections. Water SA.

[33] Zaslavsky B.Y., Miheeva L.M., Gulaeva N.D., Borovskaya A.A., Rubtsov M.I., Lukatskaya L.L., Mchedlov-Petrossyan N.O. (1991). Influence of non-ionic polymers on solvent properties of water as detected by studies of acid–base equilibria of sulphonephthalein and fluorescein dyes. J. Chem. Soc. Faraday Trans..

[34] Lippert E. (1957). Spektroskopische Bestimmung des Dipol-momentes aromatischer Verbindungen im ersten angeregten Singulettzustand. Z. Elektrochem..

[35] Klonis N., Sawyer W.H. (2000). Effect of solvent-water mixtures on the prototropic equilibria of fluorescein and on the spectral properties of the monoanion. Photochem. Photobiol..

[36] Naderi F., Farajtabar A. (2016). Solvatochromism of fluorescein in aqueous aprotic solvents. J. Mol. Liq..

[37] Timasheff S.N. (2002). Protein-solvent preferential interactions, protein hydration, and the modulation of biochemical reactions by solvent components. Proc. Natl. Acad. Sci. USA.

[38] Gerasimova M.A., Tomilin F.N., Malyar E.Y., Varganov S.A., Fedorov D.G., Ovchinnikov S.G., Slyusareva E.A. (2020). Fluorescence and photoinduced proton transfer in the protolytic forms of fluorescein: Experimental and computational study. Dyes Pigm..

[39] Alvarez-Pez J.M., Ballesteros L., Talavera E., Yguerabide J. (2001). Fluorescein excited-state proton exchange reactions: Nanosecond emission kinetics and correlation with steady-state fluorescence intensity. J. Phys. Chem. A.

[40] Magde D., Wong R., Seybold P.G. (2002). Fluorescence quantum yields and their relation to lifetimes of rhodamine 6G and fluorescein in nine solvents: Improved absolute standards for quantum yields. Photochem. Photobiol..

[41] Laage D., Stirnemann G., Sterpone F., Rey R., Hynes J.T. (2011). Reorientation and allied dynamics in water and aqueous solutions. Annu. Rev. Phys. Chem..

[42] Bonn M., Bakker H.J., Rago G., Pouzy F., Siekierzycka J.R., Brouwer A.M., Bonn D. (2009). Suppression of proton mobility by hydrophobic hydration. J. Am. Chem. Soc..

[43] Huppert D., Kolodney E., Gutman M., Nachliel E. (1982). Effect of water activity on the rate of proton dissociation. J. Am. Chem. Soc..

